# Chronic pain: prevalence, demographic inequalities, and healthcare utilisation. A primary care database analysis

**DOI:** 10.3399/BJGPO.2024.0147

**Published:** 2025-07-30

**Authors:** Siddesh Shetty, James Scuffell, Dianne Aitken, Mark Ashworth

**Affiliations:** 1 School of Life Course and Population Sciences, King’s College London, London, UK; 2 Lambeth Together Care Partnership Board, NHS Lambeth Together, London, UK

**Keywords:** pain, inequalities, health inequities, primary healthcare, general practitioners

## Abstract

**Background:**

Chronic pain (CP) is an ill-defined condition, often under-recorded in primary care records.

**Aim:**

To determine prevalence, evidence of health inequalities, primary care consultation rates, and healthcare utilisation costs of CP.

**Design & setting:**

Cross-sectional, retrospective study using anonymised primary care data from all GP practices in one inner-city London area.

**Method:**

CP was defined on the basis of analgesic medication codes and novel inclusion of diagnostic codes for conditions known to be strongly associated with CP. CP prevalence and consultation rates were determined; comparisons were made with 31 other long-term conditions (LTCs). Consultation cost estimates were based on healthcare professional type and consultation mode.

**Results:**

In total, 358 889 adult patients were registered in sample practices, with continuous (12-month) healthcare records available for 327 800 (91.3%). CP prevalence was 18.6%; the second most prevalent LTC after anxiety at 21.4%. CP mean annual consultation rates were 15.3 per patient, the highest LTC consultation rate. CP incurred the highest primary care consultation costs of any of the included LTCs. Age was the strongest predictor of CP, particularly in those aged ≥60 years (adjusted odds ratio [AOR] for 60-69 years: 9.32; 95% confidence interval [CI] = 8.83 to 9.84; compared with those aged 18–29 years). Much smaller AORs were found for ethnicity, social deprivation, sex, and non-UK country of birth.

**Conclusion:**

CP represents the most demanding LTC, in terms of consultation rates and costs, managed in primary care. Yet there is considerable uncertainty about optimal management and alternatives to long-term, high-volume primary care consultation rates.

## How this fits in

This study analysed primary care records from more than 300 000 individuals in South London, creating a chronic pain (CP) register using both medication and diagnostic codes. It found that 18.6% of patients live with CP. Compared with other long-term conditions (LTCs), people with CP had the highest primary care consultation rates and associated costs. This study highlights an important opportunity to explore alternative models of care for managing CP.

## Introduction

Chronic pain (CP) is an ill-defined condition without common consensus on coding in primary care electronic health records. Unlike long-term conditions (LTCs) included in the Quality and Outcomes Framework (QOF), which are clearly defined, there is considerable debate about inclusion thresholds for a diagnostic label of CP.^1^

CP is usually defined as pain lasting more than 3 months^2^ and has several causes. Sometimes CP is a feature of an underlying LTC such as osteoarthritis, endometriosis, or cancer (chronic secondary pain). In other cases, CP has no clear underlying cause such as fibromyalgia, chronic daily headache, irritable bowel syndrome (chronic primary pain), conditions likely to have a basis in a mix of physiological, psychological, and social factors. Approximately, one-third of people with CP have a musculoskeletal (MSK) cause, one-third report no underlying LTC, and 15% have a mental health disorder, with smaller proportions reporting less common underlying causes.^3^ Mental health and social problems may be linked both to causes and consequences.^4^

Prevalence estimates for CP vary widely with the highest levels found in community surveys, such as the Health Survey for England,^3^ which reported that 34% of adults had CP rising to 53% of those aged ≥75 years.^3^ Estimates derived from a primary care population are likely to produce lower prevalence rates than community surveys partly because research definitions are often based on use of repeat prescription records for analgesic medication rather than the inclusion of clinical diagnostic codes, thus excluding those who opt for non-drug management of chronic pain. A proportion of patients with CP are likely to self-manage and, in common with many illnesses such as anxiety or depression, not report to their GP and thus not appear in prevalence statistics.

In a UK study, using Clinical Practice Research Datalink (CPRD) data covering 400 000 adults registered in primary care, the prevalence of CP was 10.1%.^5^ Identification of patients with CP in this study relied on computerised searches for analgesic medication: four or more prescription-only medications (POMs) in the past 12 months with exclusions for analgesics with dual indication such as epilepsy (in patients with an ‘epilepsy’ coding). Using similar search criteria except for the inclusion of children or adolescents, a study from Scotland covering 1.75 million patients reported a prevalence of 7.2% for ‘painful condition’.^6^

CP is also associated with high primary care consultation rates^7,8^ with an estimated 22% of all primary care consultations (in Finland) attributable to CP.^9^ However, rates have not been clearly defined for UK primary care populations.^10^ High prevalence combined with high consultation rates indicates high primary care utilisation rates and costs. There is evidence of high cost associated with specified conditions, such as osteoarthritis,^11^ with data obtained from relatively small patient survey sample data^12^ but not based on broader ‘chronic pain’ categories.

Demographic data on CP prevalence indicates that there are considerable health inequalities related to age, sex, social deprivation, and ethnicity.^3,13,14^ Prevalence is higher in more deprived communities and also in ethnic minority groups. If people with CP are high users of primary care, there may be opportunities to support culturally appropriate alternatives to GP consultations.

We aimed to analyse CP prevalence, health inequalities, primary care consultation rates, and healthcare utilisation costs based on primary care data from an urban, deprived, and multi-ethnic community.

## Method

### Definition of chronic pain

We used a novel and inclusive definition of CP to create a CP register, applicable to anonymised, coded primary care datasets.^15^ This definition is based on both medication and diagnostic codes. In line with previous CP studies in primary care,^5^ medication codes identify patients with four or more prescription-only analgesics in the previous 12 months, including tricyclic antidepressants and anti-epileptic medication when used for pain control. Diagnostic codes identify patients with one or more of 33 conditions known to be associated with CP but excluding cancer pain (although including ‘pathological fracture’). A hierarchy of conditions was constructed such that patients with conditions strongly associated with pain were included even without evidence of associated analgesic prescriptions, whereas those with conditions less strongly associated with CP were only included if there was evidence of at least three prescription-only analgesics in the previous 12 months. Patients may be included on the CP register based on either their medication or diagnostic categories, or both. The codes used were based on a scoping review of painful conditions.^15^

### Study design, setting, participants, and data

This study was a cross-sectional, retrospective database study using anonymised primary care data. We based our analysis on the previously used algorithm to identify patients with CP;^15^ for comparison, we included 31 other LTCs using a method defined in a previous study.^16^

Primary care data were obtained from Lambeth DataNet, an anonymised database containing coded data (SNOMED-CT and EMIS demographic and medication and devices codes) from all patients aged ≥18 years registered at all GP practices in one inner London borough (Lambeth), except for those with ‘informed dissent’ codes in their electronic health record (2.6%).^17–19^ Data extraction was conducted on 31 August 2023. Patients were excluded from the analysis if they did not have a whole calendar year of data before data extraction. The database contains detailed ethnicity, country of origin, and language preference data. Ethnicity coding was based on the five national Census codes^20^ with more detailed ethnicity data available using EMIS demographic codes. Social deprivation data were based on the Index of Multiple Deprivation (IMD) 2019, stratified into local quintiles.^21^

### Statistical methods

Raw CP and comparator LTC prevalence data, categorised by demographic characteristics, were summarised as descriptive statistics. Similarly, annual consultation rate data were summarised according to consultation type and mode.

Health economic analysis was based on CP primary care consultation rates and comparator LTCs categorised by type of care provider (GP, nurse, other healthcare professional) and by consultation mode (face to face, telephone, home visit, digital). National unit costs from the year 2022 were calculated for each consultation type (provider and mode), adjusted for mean consultation duration, based on Personal Social Services Research Unit estimates.^22,23^

Finally, multivariable binary logistic regression modelling was used to determine the strength of association between CP and 10-year age bands, sex, IMD quintiles, country of birth, language preference, and the 5+1 Census ethnic groups plus Black African and Black Caribbean. Complete case analyses were used.

## Results

### Study sample

A total of 358 889 adult patients were registered at the sample practices, with continuous healthcare records available over a 12-month period for 327 800 (91.3%). All analyses were conducted on this final sample. Ethnicity was known in 302 640 (92.3%), of whom 177 084 (58.5%) were White ethnicity, 65 942 (21.8%) were Black ethnicity, 26 687 (8.8%) were Asian ethnicity, 17 772 (5.9%) were Mixed ethnicity, and 15 155 (5.0%) were of Other ethnicity. Of the Black ethnicity population, data were available to categorise either as Black African ethnicity (33 313, 11.0%) or Black Caribbean ethnicity (20 942, 6.9%). Language preference data were available for 248 851 (75.9%), of whom 178 012 (71.5%) were English speakers and 70 839 (28.5%) were non-English speakers, by ‘preference’. Similarly, country of origin data were available for 177 554 (54.2%), of whom 80 242 (45.2%) were born in the UK and 97 292 (54.8%) were born outside the UK. Social deprivation data (IMD-2019 scores) were categorised as within-borough quintiles, resulting in equal population distribution between each quintile. Mean consultation rate was 6.7 consultations per person per year.

### Chronic pain prevalence

**Figure 1.**
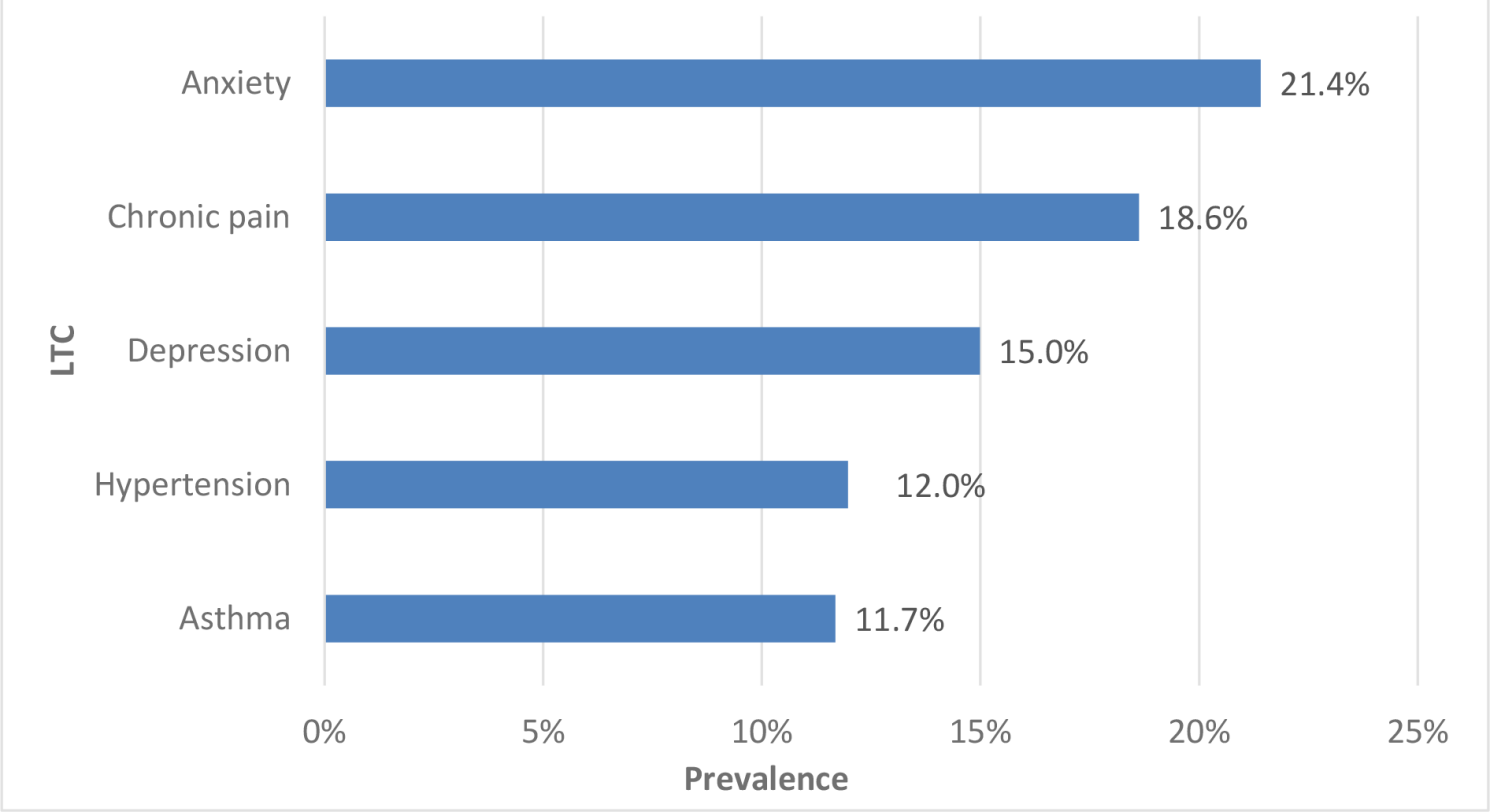
Highest ranked long-term conditions based on reported prevalence. LTC = long-term condition

The overall prevalence of CP was 18.6%. CP was the second most prevalent LTC based on LTCs included in our study; the highest prevalence was for anxiety, 21.4% (Figure 1).

There were large differences in CP prevalence according to demographic categories. For example, CP prevalence was 13.6% in males and 24% in females; 17.5% in White ethnicity patients and 27.2% in Black ethnicity patients. Notably, prevalence was higher in Black Caribbean than Black African patients (32.4% versus 25.1%, respectively). In a post-hoc analysis, it appeared that this difference was likely driven by age differences between the two groups; the mean age of the Black Caribbean group was 49.9 years, compared with 45.1 years in the Black African group (*t* = 31.7; *P*<0.001); and a higher proportion of people aged over 65, 20.3% versus 11.3%, respectively (*t* = 14.9; *P*<0.001). Further details are described in Supplementary Tables S1, S2, and S3.

### Chronic pain primary care consultation rates

**Figure 2.**
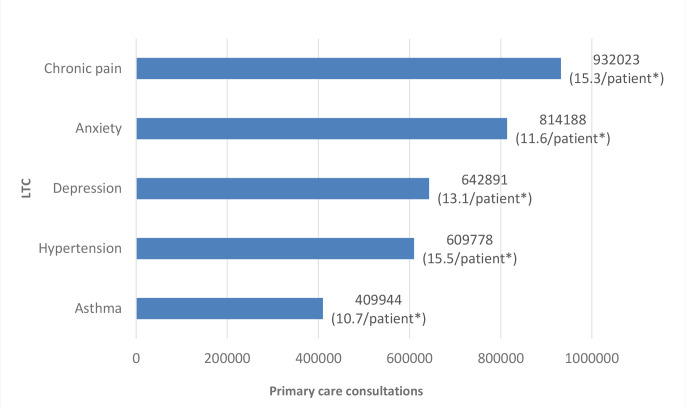
Highest ranked long-term conditions based on annual primary care consultation rates and total consultation numbers. *Number of consultations per patient per year for people living with each listed long-term condition

The overall mean annual consultation rate for patients with CP was 15.3, accounting for 932 023 primary care consultations, the highest annual consultations for any of the included LTCs. The next most frequent LTC in terms of consultation numbers was anxiety, accounting for 814 188 consultations with each patient consulting a mean of 11.6 times annually (Figure 2 and Supplementary Figure S1).

### Chronic pain primary care costs

**Figure 3.**
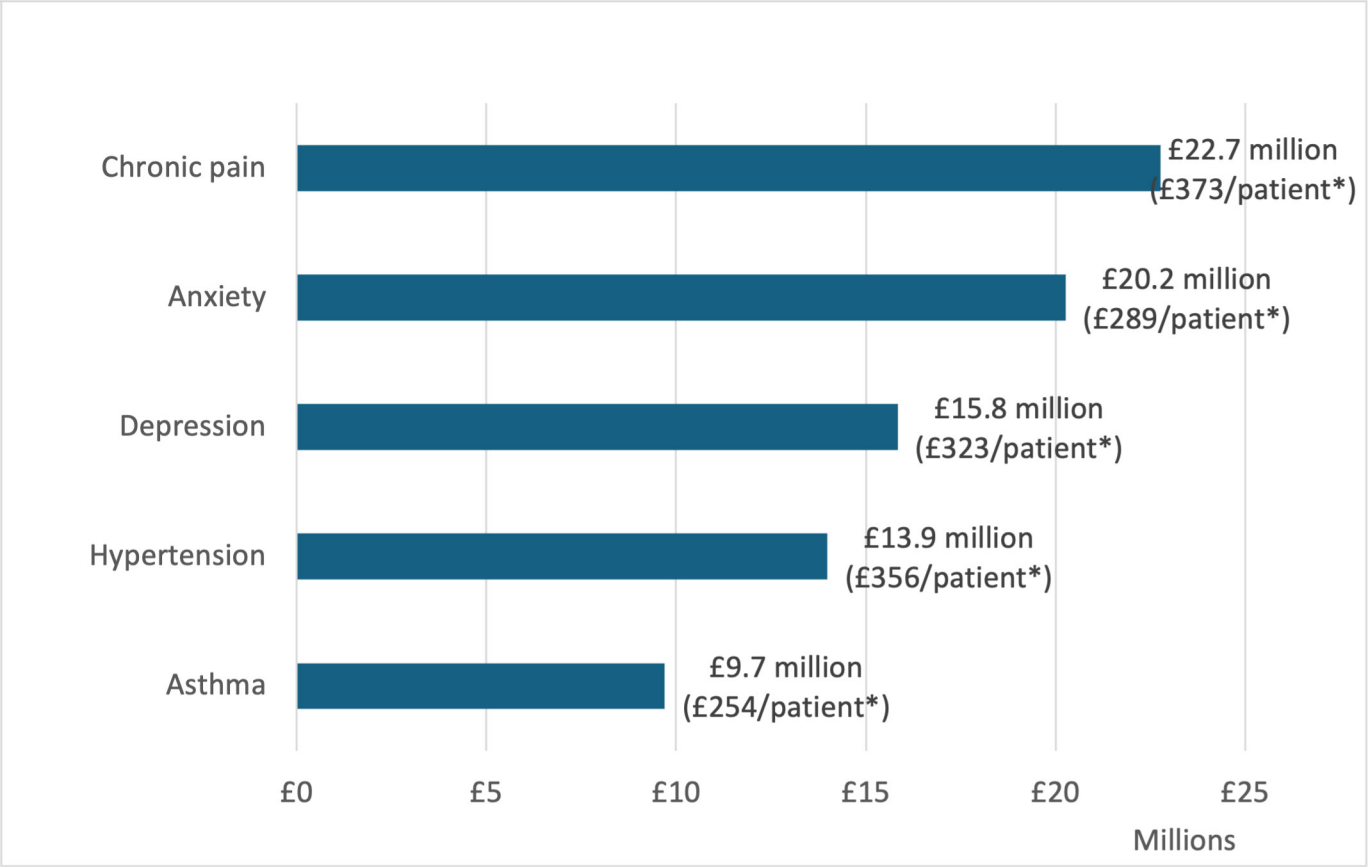
Highest ranked long-term conditions (LTC) based on total annual primary care consultation costs, all patients. *Annual cost per patient per year in GBP for people living with each listed long-term condition. The breakdown of these costs are reported in Table 1.

CP was also the highest cost condition based on annual primary care costs, categorised by both consultation provider and consultation mode. The total cost was £22.7 million, equating to £373 per patient. The next most costly condition was anxiety with a total cost of £20.2 million, equating to £289 per patient (Figure 3, Table 1)

**Table 1.** Summary of annual consultation costs and consultation provider for highest-cost long-term conditions (LTCs)

Long-term condition	Total cost	GP consultation cost	Nurse consultation cost	Other healthcare provider cost
**Chronic pain** (***n* = 61 042**)	£22.7 million (£373^a^)	£19.1 million (£313^a^)	£1.4 million (£23^a^)	£2.2 million (£37^a^)
**Anxiety** **(*n* = 70 181)**	£20.2 million (£289^a^)	£17.2 million (£245^a^)	£1.1 million (£17^a^)	£1.8 million (£26^a^)
**Depression** **(*n* = 49 113)**	£15.8 million (£323^a^)	£13.3 million (£273^a^)	£0.9 million (£19^a^)	£1.5 million (£31^a^)
**Hypertension** **(*n* = 39 247)**	£13.9 million (£356^a^)	£11.2 million (£286^a^)	£1.0 million (£27^a^)	£1.6 million (£43^a^)
**Asthma** **(*n* = 38 281)**	£9.7 million (£254^a^)	£7.8 million (£204^a^)	£0.8 million (£21^a^)	£1.0 million (£28^a^)
**CP prevalence**	**White ethnicity**	**Black Caribbean ethnicity**	**Black African ethnicity**	**Asian ethnicity**
**Overall**	£11.0 million (£356^a^)	£2.9 million (£436^a^)	£3.1 million (£371^a^)	£1.7 million (£396^a^)
**Male**	£3.7 million (£312^a^)	£0.7 million (£361^a^)	£0.9 million (£328^a^)	£0.6 million (£346^a^)
**Female**	£7.2 million (£385^a^)	£2.1 million (£473^a^)	£2.1 million (£394^a^)	£1.1 million (£432^a^)
**Born in UK**	£4.6 million (£373^a^)	£0.7 million (£433^a^)	£0.2 million (£381^a^)	£0.1 million (£388^a^)
**Born outside UK**	£2.0 million (£343^a^)	£1.0 million (£445^a^)	£1.5 million (£373^a^)	£0.8 million (£405^a^)
**English language preference**	£7.6 million (£358^a^)	£2.4 million (£442^a^)	£1.5 million (£374^a^)	£0.7 million (£416^a^)
**Non-English language preference**	£1.6 million (£345^a^)	£0.03 million (£419^a^)	£0.7 million (£368^a^)	£0.7 million (£391^a^)

^a^Annual cost per patient per year in GBP for people living with each listed long-term condition and consultation provider. CP = chronic pain

### Demographic predictors of chronic pain prevalence: binary logistic regression

We explored strength of independent association between CP prevalence and demographic factors using binary logistic regression. Outcomes are presented as adjusted odds ratios (aOR) with 95% confidence intervals (Table 2). After adjustment, age remained the strongest predictor of CP, with those aged 60-69 nine times more likely to have CP than those aged 18-29 (aOR 9.32, 95% CI 8.83–9.84).

**Table 2.** Adjusted odds ratios and 95% confidence intervals for chronic pain (outcome) and demographic factors (independent variables)

Demographic factors	Adjusted odds ratio	95% confidence interval, Adjusted odds ratio
**Age: 18–29 years**	REFERENCE GROUP	
**Age: 30–39 years**	1.67	1.59 to 1.76
**Age: 40–49 years**	3.07	2.92 to 3.23
**Age: 50–59 years**	5.61	5.34 to 5.90
**Age: 60–69 years**	9.32	8.83 to 9.84
**Age: 70–79 years**	14.58	13.68 to 15.54
**Age: 80–89 years**	27.73	25.46 to 30.20
**Age: ≥90 years**	39.91	33.74 to 47.21
**Sex: male**	REFERENCE GROUP	
**Sex: female**	2.22	2.16 to 2.28
**Social deprivation:** **quintile: 1 (least deprived)**	REFERENCE GROUP	
**Social deprivation:** **quintile: 2**	1.16	1.11 to 1.22
**Social deprivation:** **quintile: 3**	1.28	1.23 to 1.34
**Social deprivation:** **quintile: 4**	1.56	1.60 to 1.64
**Social deprivation:** **quintile: 5 (most deprived)**	1.70	1.63 to 1.78
**Ethnicity: White**	REFERENCE GROUP	
**Ethnicity: Black Caribbean**	1.51	1.44 to 1.59
**Ethnicity: Black African**	1.49	1.43 to 1.56
**Ethnicity: Asian**	1.16	1.10 to 1.22
**Ethnicity: Mixed**	1.30	1.23 to 1.37
**Ethnicity: Other**	1.06	0.99 to 1.13
**Ethnicity: recorded as ‘unknown’**	0.87	0.79 to 0.96
**Language preference:** **English**	REFERENCE GROUP	
**Language preference:** **non-English**	0.95	0.92 to 0.99
**Country of birth:** **UK**	REFERENCE GROUP	
**Country of birth:** **non-UK**	0.61	0.59 to 0.63

## Discussion

### Summary

CP is a high prevalence (18.6%), high consultation rate (15.3 per patient per year) condition in primary care. The combination of high prevalence and high consultation rates drives high costs. CP was the single highest cost LTC with an annual consultation cost of £373 per patient per year. This equates to 29% higher costs than the second most costly condition, anxiety. We identified several demographic factors associated with CP prevalence, but the dominant factor was age to a far greater extent than sex, social deprivation, ethnicity, and non-UK country of origin, although these remain important factors.

### Strengths and limitations

The novel method used in this study to determine the adult prevalence of CP will have captured patients with CP but not taking any regular analgesics, as per National Institute for Health and Care Excellence (NICE) guidance.^3^ On the other hand, CP prevalence may have been artificially inflated by the presence of known painful diagnoses for which pain was only an intermittent rather than chronic feature. In mitigation, only 12 of the 33 included LTCs associated with pain triggered a diagnosis of CP based on clinical diagnostic coding alone (so-called ‘Tier 1’ conditions), without concurrent analgesic prescription.^15^

We did not access coding labels for each primary care consultation, as these are not reliably recorded for all consultations. As a result, our data summarised overall consultation rates for patients with CP, and not pain-specific consultations. It is therefore probable that a proportion of these consultations were unrelated to CP, and may have been attributable to comorbidities, of which depression and anxiety are common.^22^ Moreover, primary care costs are likely to be dominated not just by individual LTCs, but by LTCs commonly associated with CP.^22^ However, it seems plausible that a holistic approach to CP management may have a more generalised effect on health-seeking behaviour for other LTCs. Future work should explore CP-specific consultations, considering practice- and clinician-level differences in recording. An evidence base for the impact of pain clinic referrals on primary care utilisation is also needed.

Cost estimates were based on predicted consultation duration and healthcare professional type. They did not include medication costs, which may vary considerably between patients with CP, nor did they include social care or secondary care costs, which will be additional, although there may be an element of complementarity or substitution.^24^

Cross-sectional analysis (univariable) appeared to show large differences in CP prevalence, which were not borne out by multivariable analysis. For example, univariable analysis demonstrated a higher CP prevalence in Black Caribbean compared with Black African patients (32.4% versus 25.1%, respectively).

### Comparison with existing literature

Based on this novel method of capturing a diagnosis of CP, using both medication and diagnostic codes, the reported CP prevalence was substantially higher in our study than comparable primary care studies using only medication codes.^5^ However, in our study, the population was not representative of the UK population having the typical features of an inner-city, multi-ethnic deprived community, with a much higher proportion of Black ethnicity and socially deprived population; on the other hand, the sample population was much younger, and therefore expected to have lower CP prevalence.^25^

The strong association between CP prevalence and age is borne out by several studies,^3,13,26^ especially advanced age.^26^ In one European study, 70% of those aged ≥90 years had moderate or severe pain.^27^ The association with deprivation^26^ and ethnicity, particularly with Black ethnicity,^13^ has been previously described in health surveys. Our study adds that people of Black ethnicity with CP also have higher primary care usage than other ethnic groups. This may be owing to underlying differences in prevalence, or that CP in the Black ethnic group is more associated with disability and depression,^14^ which may drive higher primary care use. Previous reports have not highlighted the non-UK country of origin, which in our study was associated with lower CP prevalence, possibly because of a ’healthy migrant’ effect^28^ or differential utilisation of primary care by migration status. After adjustment there were only small disparities in CP prevalence by language preference.

### Implications for research and practice

There is a workload crisis in UK primary care at present and CP represents the most demanding LTC (in terms of consultation rates and costs) managed in primary care. Yet there is considerable uncertainty about optimal CP management and what alternatives there are to long-term, high-volume primary care consultation rates.^2^ There is an opportunity to co-design services using a bio-psycho-social model, to better support the needs of minoritised populations.^29^ Implementation studies are urgently needed to determine alternatives to GP consultations (by far the largest proportion of all CP consultations in our study). Alternatives to primary care are needed but have generally been poorly evaluated, such as management by social prescribers, psychologists, physiotherapists, pharmacists, or self-management approaches.^26^ To make changes in practice, it is important to be able to both reach a common definition of CP in primary care and identify these patients through creation of a disease register. In current UK practice, most disease registers are focused on QOF LTCs, which currently excludes CP and concerns have been raised about negative effects on care and lack of coding for non-incentivised LTCs such as CP.^30,31^ Incorporating a CP disease register into standard UK general practice would aid primary care to better measure and evaluate interventions to reduce GP workload.

## Data

The code lists and logic rules developed during the current study are available from the corresponding author on reasonable request.
